# Inhibition of Protein Prenylation of GTPases Alters Endothelial Barrier Function

**DOI:** 10.3390/ijms21010002

**Published:** 2019-12-18

**Authors:** Muhammad Aslam, Christian Troidl, Christian Tanislav, Susanne Rohrbach, Dursun Gündüz, Christian W. Hamm

**Affiliations:** 1 Department of Cardiology/Angiology, University Hospital Giessen, 35392 Giessen, Germany; christian.troidl@innere.med.uni-giessen.de (C.T.); dursun.guenduez@innere.med.uni-giessen.de (D.G.); christian.hamm@innere.med.uni-giessen.de (C.W.H.); 2 Experimental Cardiology, Justus Liebig University, 35392 Giessen, Germany; 3 DZHK (German Centre for Cardiovascular Research), partner site Rhein-Main, 61231 Bad Nauheim, Germany; 4 Department of Neurology, Evangelisches Jung Stilling Krankenhaus GmbH, 57074 Siegen, Germany; Christian.Tanislav@neuro.med.uni-giessen.de; 5 Institute of Physiology, Justus Liebig University, 35392 Giessen, Germany; Susanne.Rohrbach@physiologie.med.uni-giessen.de; 6 Department of Cardiology and Angiology Evangelisches Jung Stilling Krankenhaus GmbH, 57074 Siegen, Germany

**Keywords:** Rac1, peripheral actin, statins, geranylgeranyl transferase, endothelial permeability, RhoA, adherens junctions

## Abstract

The members of Rho family of GTPases, RhoA and Rac1 regulate endothelial cytoskeleton dynamics and hence barrier integrity. The spatial activities of these GTPases are regulated by post-translational prenylation. In the present study, we investigated the effect of prenylation inhibition on the endothelial cytoskeleton and barrier properties. The study was carried out in human umbilical vein endothelial cells (HUVEC) and protein prenylation is manipulated with various pharmacological inhibitors. Inhibition of either complete prenylation using statins or specifically geranylgeranylation but not farnesylation has a biphasic effect on HUVEC cytoskeleton and permeability. Short-term treatment inhibits the spatial activity of RhoA/Rho kinase (Rock) to actin cytoskeleton resulting in adherens junctions (AJ) stabilization and ameliorates thrombin-induced barrier disruption whereas long-term inhibition results in collapse of endothelial cytoskeleton leading to increased basal permeability. These effects are reversed by supplementing the cells with geranylgeranyl but not farnesyl pyrophosphate. Moreover, long-term inhibition of protein prenylation results in basal hyper activation of RhoA/Rock signaling that is antagonized by a specific Rock inhibitor or an activation of cAMP signaling. In conclusion, inhibition of geranylgeranylation in endothelial cells (ECs) exerts biphasic effect on endothelial barrier properties. Short-term inhibition stabilizes AJs and hence barrier function whereas long-term treatment results in disruption of barrier properties.

## 1. Introduction

The integrity of the vascular endothelial (VE) barrier is maintained by the equilibrium of competing contractile and adhesive forces generated by the acto-myosin-based endothelial cell (EC) contractile machinery and adhesive molecules located at cell–cell and cell–matrix contacts, respectively [1]. ECs are tightly connected with each other by the interaction of adherens junction (AJ) proteins (VE-cadherin and catenins) of adjacent cells, which are linked to the peripheral actin cytoskeleton present directly under the cell membrane [2,3]. Therefore, changes in the activation state of the EC contractile machinery and/or actin cytoskeleton dynamics affect the stability of EC AJs.

The Rho family of GTPases, particularly RhoA and Rac1, are the major regulators of EC contraction, actin cytoskeleton dynamics, and AJs and hence play a pivotal role in the maintenance of EC barrier integrity. Overexpression of a constitutive active RhoA resulted in the loss of basal VE-cadherin and potentiated the effect of hypoxia-reoxygenation (H/R) whereas overexpression of dominant negative RhoA attenuated the agonist-induced endothelial hyperpermeability [4,5]. On the other hand, overexpression of constitutively inactive Rac1 in cultured ECs resulted in increased endothelial permeability and abolished the recovery of endothelial barrier function following H/R-mediated barrier failure. Accordingly, overexpression of a constitutively active form of Rac1 showed a strong junctional staining of VE-cadherin and abolished H/R-induced loss of cell–cell contacts [4]. Unlike RhoA and Rac1, overexpression of a constitutively active or inactive form of Cdc42 had no significant effect on endothelial barrier integrity [4,6,7,8]. These GTPases act as molecular switches and their activation is primarily regulated by shuttling between the GDP- (inactive) or the GTP-bound (active) form [9]. Besides, switching between GDP and GTP form, their spatial localization and activity is regulated by post-translational lipidation or prenylation at the CAAX (Cys-aliphatic-aliphatic-X) motif [10]. The prenylation of GTPases can be blocked by HMG-CoA reductase inhibitors (statins) leading to reduced production of the substrate or more specifically downstream enzymes responsible for the addition of these moieties to the GTPases [11].

In the present study we investigated the effects of short- and long-term inhibition of protein prenylation on endothelial AJs and barrier function using an in vitro cell culture model of human umbilical cord endothelial cells (HUVECs). We report here a biphasic effect of short- and long-term inhibition of protein prenylation on HUVEC permeability. Short-term treatment has EC barrier protective effects whereas long-term inhibition has barrier disrupting effects.

## 2. Results

### 2.1. Statins Suppress Endothelial Barrier Function

**Figure 1:**
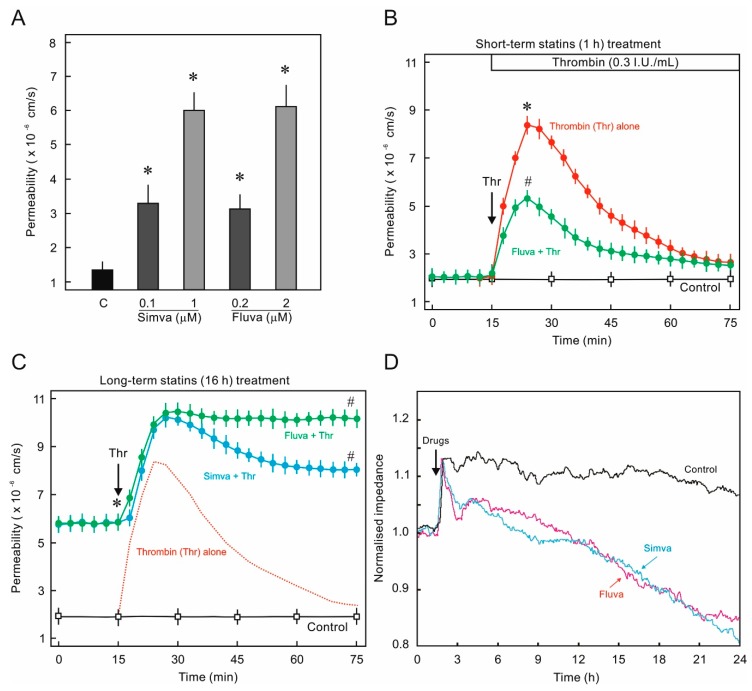
Effect of statins on human umbilical vein endothelial cells (HUVEC) monolayer permeability. (**A**) Conc.-dependent effect of the statins on HUVEC permeability. HUVEC monolayers were treated with various concentrations of simvastatin (simva) and fluvastatin (fluva) for 16 h as indicated, and the albumin permeability was measured. The graph shows albumin flux rate after 3 h measurement. *n* = 5; *p* < 0.05; * vs. control. (**B**) Short-term statin treatment effect on HUVEC permeability. HUVECs were pretreated with fluvastatin (Fluva; 2 μM) or buffer (control) for 1 h and permeability was measured. After a stabilization period of 15–30 min, thrombin (Thr) was added as indicated (arrow). *n* = 5; *p* < 0.05; * vs. control, ^#^ vs. Thr alone. (**C**) Long-term statin treatment effect on HUVEC permeability. HUVECs were pretreated with simvastatin (Simva; 1 μM), fluvastatin (Fluva; 2 μM) or buffer (control) for 16 h and albumin permeability was measured. After a stabilization period of 15–30 min, thrombin (Thr) was added as indicated (arrow). The dotted red line indicates typical reaction of HUVEC to thrombin. *n* = 5; *p* < 0.05; * vs. control, ^#^ vs. Thr alone. (**D**) Effect of statins on HUVEC trans-endothelial electrical resistance (TEER). HUVEC were cultured on gold-coated electrodes until confluence and afterwards treated with simvastatin, fluvastatin, or DMSO (control) as indicated (arrow) and TEER was measured continuously for further 24 h. The tracing shows representative (of three independent experiments) normalized impedance (TEER) of HUVEC monolayers.

In the first instance the effect of long-term treatment of statins on EC barrier properties was investigated. We used two chemically different statins and both caused a two-fold increase in permeability already at low concentrations (0.1–0.2 µM) and four-fold increase at medium concentrations (1–2 μM) (Figure 1A). In-contrast, short-term (1 h) treatment of statins has no effect on basal permeability but antagonized thrombin-induced EC hyper-permeability (Figure 1B). Thrombin causes a transient and reversible increase in EC permeability which returns to basal level within an hour after the challenge [12,13]. However, when the cells were pretreated with statins for 16 h, this transient effect of thrombin on EC permeability was lost and thrombin-induced hyperpermeability persisted during whole period of measurement and it did not return to the initial basal state (Figure 1C). The barrier disrupting effect of long-term statin treatment was reproduced by using a different (impedance-based) model of EC barrier properties analyses. In this model, a reduction in impedance represents a leaky EC monolayer. Statins caused a consistent decrease in impedance over a period of 24 h (Figure 1D).

**Figure 2:**
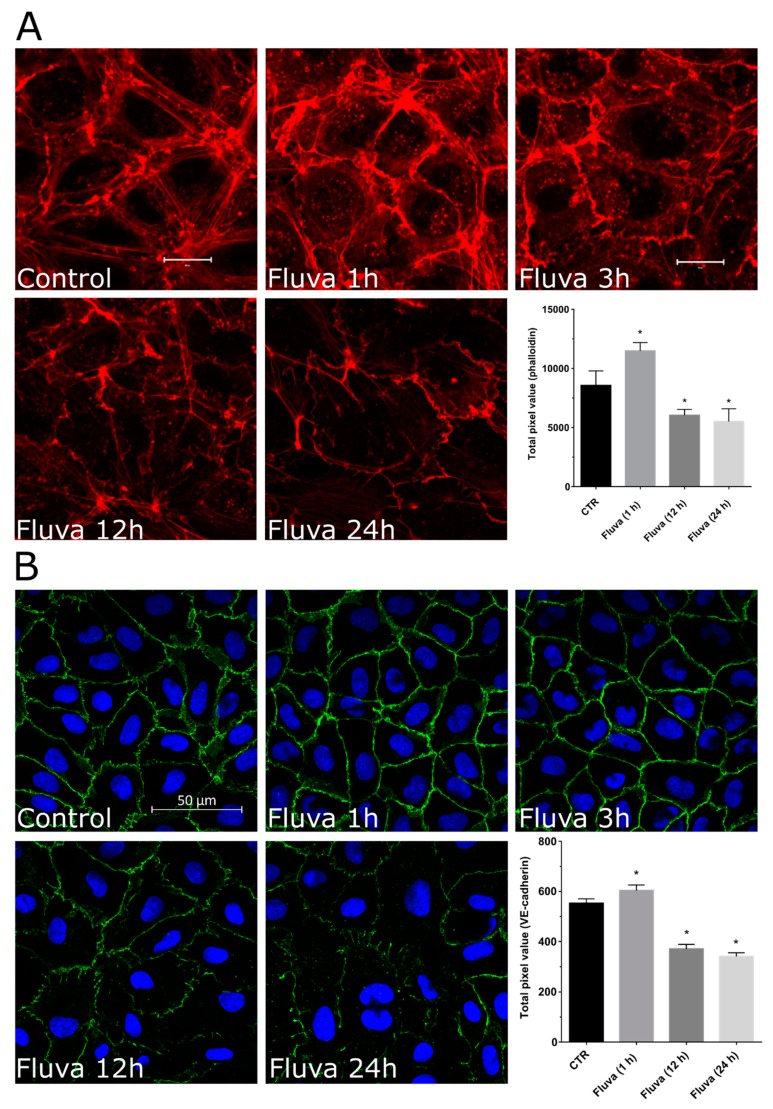
Effect of statins on HUVEC actin cytoskeleton and vascular endothelial (VE)-cadherin. (**A**) Time-dependent effect of fluvastatin on actin cytoskeleton. HUVECs were cultured on coverslips until confluence and treated with fluvastatin (2 μM) for indicated time or buffer (control; C). Representative immunofluorescence images (from five independent experiments) of F-actin labelled with phalloidin-TRITC. Scale bar: 20 μm. The graph shows pixel values of phalloidin from 3–5 images. *n* = 3; *p* < 0.05; * vs. control. (**B**) Time-dependent effect of fluvastatin on VE-cadherin. HUVECs were cultured on coverslips until confluence and treated with fluvastatin (2 μM) for the indicated times or buffer (control; C). Representative immunofluorescence images (from five independent experiments) of VE-cadherin. Scale bar: 50 μm. The graph shows grey-scale pixel values of VE-cadherin from 3–5 images. *n* = 3; *p* < 0.05; * vs. control.

EC cytoskeleton dynamics, particularly the peripheral actin, plays a vital role in the maintenance of endothelial barrier function. Statins caused disruption of EC actin cytoskeleton in a time-dependent manner. Initially, statin treatment resulted in the loss of actin stress fibers running across the cell after 1 h of treatment, however, the peripheral actin was preserved at this stage. Later after 3 h, the peripheral actin started also disappearing and after 12 h only some random actin fibers at the cell periphery were visible (Figure 2A). The changes in actin cytoskeleton dynamics were accompanied by the changes in VE-cadherin labelling at the cell-cell junctions. After 1 h of statin treatment, relatively stronger staining of VE-cadherin was visible that was much reduced after 12–24 h of statin treatment (Figure 2B).

### 2.2. Protein Prenylation and Endothelial Barrier Properties

**Figure 3:**
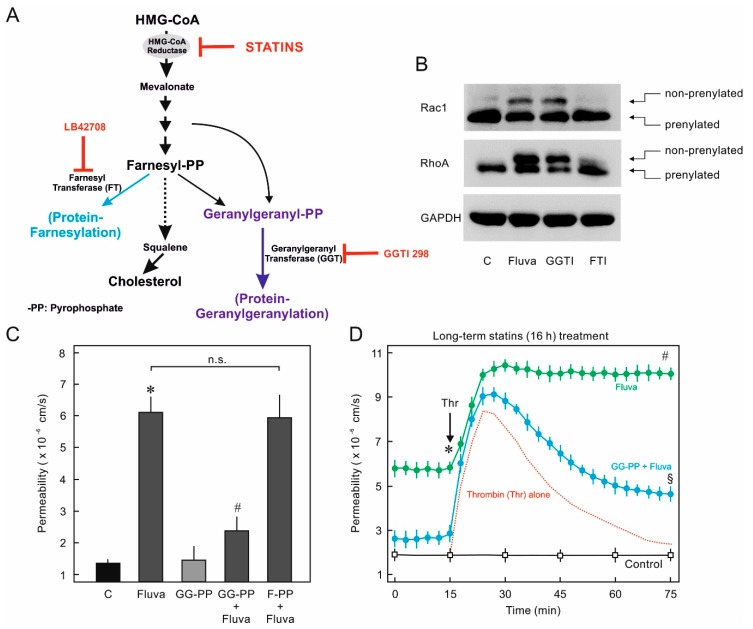
Effect of statins on prenylation of Rho GTPases. (**A**) Schematic presentation of various steps involved in protein prenylation and available pharmacological tools for intervention (Dotted and multiple arrows indicate there are multiple steps involved in between). (**B**) Effect of statins and other prenylation inhibitors on RhoA and Rac1 prenylation. HUVEC were treated with fluvastatin (2 μM), geranylgeranyl transferase inhibitor (GGTI; 10 μM), farnesyl transferase inhibitor (FTI, LB42708; 10 μM), or DMSO (C; control) for 16 h. The cells were lysed in prenylation processing buffer and subjected to Western blot analysis. The upper band represents the nonprenylated form of the GTPases. Representative blots from three experiments. (**C**) Reversal of statin effect with geranylgeranyl pyrophosphate (GG-PP) supplement. HUVEC monolayers were treated with fluvastatin (Fluva; 2 μM) in the absence or presence of GG-PP (2 μM) for 16 h, as indicated, and the albumin permeability was measured. The graph shows albumin flux rate after 3 h measurement. *n* = 3; *p* < 0.05; * vs. control, * vs. Fluva alone, n.s.:not significantly different (**D**) HUVEC monolayers were treated with fluvastatin (Fluva; 2 μM) in the absence or presence of GG-PP (2 μM) for 16 h as indicated and the albumin permeability was measured. After a stabilization period of 15–30 min, thrombin (Thr) was added as indicated (arrow). The dotted red line indicates a typical reaction of HUVEC to thrombin in the absence of any pharmacological agent. *n* = 5; *p* < 0.05; * vs. control, ^#^ vs. Thr alone. ^§^ vs. Fluva plus Thr.

Statins mainly target the HMG-CoA reductase and thus block the cholesterol and related byproducts biosynthesis. These byproducts are required for the prenylation (lipidation) of several cellular proteins particularly the members of small GTPase family such as RhoA and Rac1 and thus their spatial localization at the cellular membranes for proper functioning (Figure 3A). The small GTPases RhoA and Rac1 regulate EC actin cytoskeleton and, therefore, their prenylation was investigated by Western blots. The prenylated form of GTPases contain an additional lipid molecule at their C-terminus and are deficient of three terminal amino acids compared to the nonprenylated form and thus run faster in SDS-PAGE under optimized conditions and can be separated from the nonprenylated form. In order to investigate which form of prenylation RhoA and Rac1 undergo, the cells were treated with either farnesyl transferase inhibitor (FTI) or geranylgeranyl transferase inhibitor (GGTI). As demonstrated in Figure 3B, both RhoA and Rac1 were modified mainly by geranylgeranyl transferase (GGT) with the addition of geranylgeranyl group, as Rac1 and RhoA prenylation was inhibited by GGTI (GGTI 298). A minor inhibition of RhoA prenylation was observed in cells treated with FTI that may be due to its nonspecific activity. This was supported by the fact that supplementing the medium with geranylgeranyl pyrophosphate but not farnesyl pyrophosphate could rescue the HUVEC monolayer against statin-induced EC hyper-permeability (Figure 3C,D). Moreover, we did not observe changes in actin cytoskeleton after incubation with FTI as observed with statins (data not shown).

### 2.3. Biphasic Effect of GGT Inhibitor on EC Permeability and Cellular Junctions

**Figure 4:**
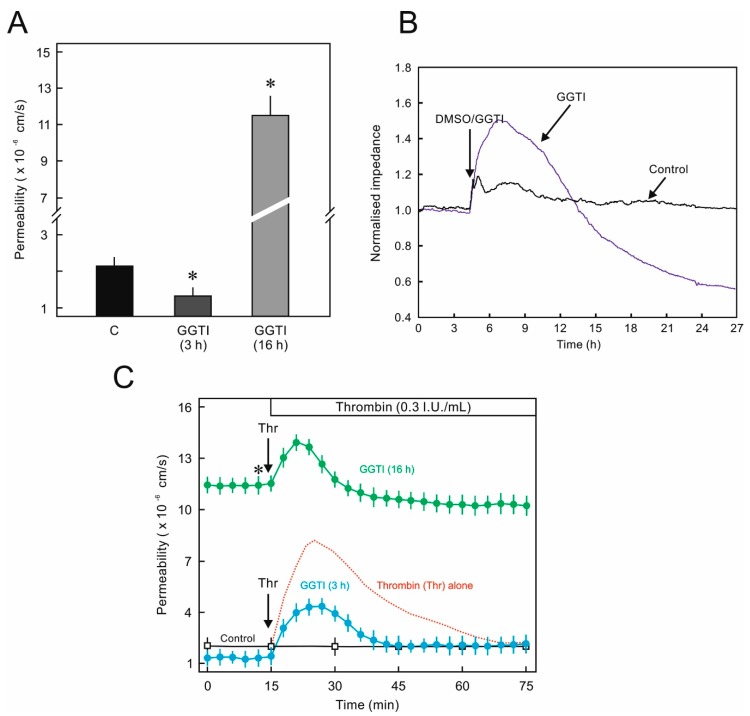
Effect of geranylgeranyl transferase (GGT) inhibition on HUVEC permeability. (**A**) Time-dependent effect of geranylgeranyl transferase inhibitor (GGTI) on endothelial cell (EC) permeability. HUVEC were treated with GGTI (10 μM) or DMSO (C; control) for indicated time periods and albumin permeability was measured. The graph shows flux rate after 3 h measurement. *n* = 5; *p* < 0.05; * vs. control. (**B**) Effect of GGTI on HUVEC trans-endothelial electrical resistance (TEER). HUVEC were cultured on gold-coated electrodes until confluence and afterwards treated with GGTI (10 μM) or DMSO (control) as indicated (arrow) and TEER was measured continuously for a further 24 h. The tracing shows representative (of three independent experiments) normalized impedance of HUVEC monolayers. (**C**) Short- vs. long-term GGTI treatment effect on HUVEC permeability. HUVECs were pretreated with GGTI (10 μM) or DMSO (control) for 3 and 16 h as indicated and albumin permeability was measured. After a stabilization period of 15–30 min, thrombin (Thr) was added as indicated (arrow). The dotted red line indicates a typical reaction of HUVEC to thrombin in the absence of any pharmacological agent. *n* = 5; *p* < 0.05; * vs. control.

**Figure 5:**
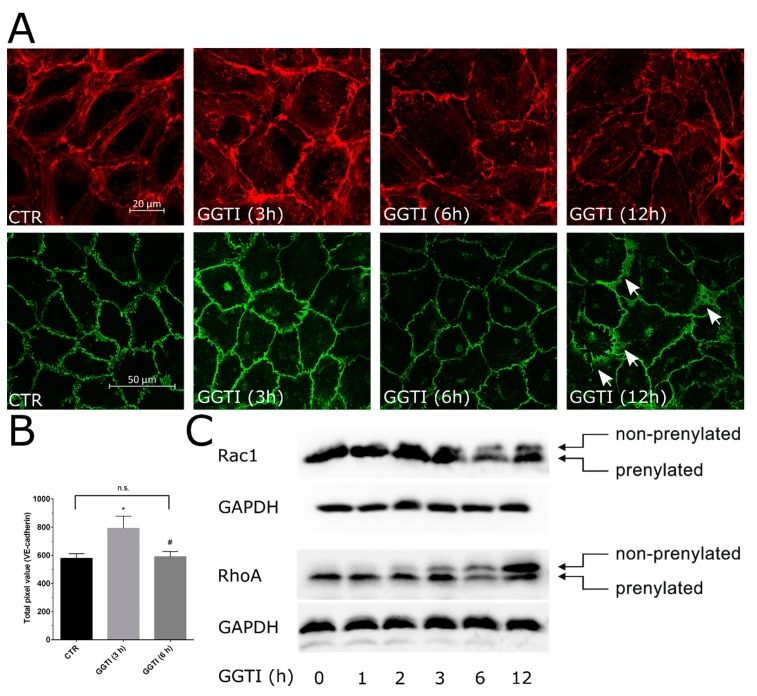
Time-dependent effect of GGT inhibition on actin cytoskeleton, cell–cell junctions and RhoA/Rac1 prenylation. (**A**) Time-dependent effect of GGTI on actin cytoskeleton and VE-cadherin. HUVECs were cultured on coverslips until confluence and treated with GGTI (10 μM) for indicated time periods or DMSO (control; C). Representative immunofluorescence images (from five independent experiments) of F-actin labelled with phalloidin-TRITC and VE-cadherin using a specific antibody. Arrows indicate reticular network like structures of VE-cadherin. Scale bar 20 or 50 μm as indicated. (**B**) The graph shows grey-scale pixel values of VE-cadherin from 3–5 images. *n* = 3; *p* < 0.05; * vs. control. (**C**) Time-dependent effect of GGTI on Rac1/RhoA prenylation. HUVEC monolayers were treated with GGTI (10 μM) for indicated time periods and analyzed for Rac1 and RhoA prenylation. Representative blots from three experiments.

In order to further investigate the effect of inhibition of prenylation of Rho GTPases, a pharmacological inhibitor of GGT was used. GGT inhibitor had a time-dependent biphasic effect on basal endothelial permeability. Within the first 3 h it reduced the permeability (Figure 4A) and caused an increase in impedance which slowly started declining and a massive reduction in impedance accompanied by an increase in macromolecular permeability was observed after 24 h (Figure 4A,B). Moreover, short-term treatment ameliorated thrombin-induced hyperpermeability (Figure 4C), however, in case of long-term treatment a similar phenomenon was observed as described above in the case of statin treatment. An investigation of the actin cytoskeleton and VE-Cadherin showed an increase in peripheral actin staining and enhanced VE-cadherin localization at cell borders after 3 h (Figure 5A,B). However, after 12 h, a massive loss of basal actin cytoskeleton was observed (Figure 5A). After 12 h of GGTI treatment, the adherens junctions changed from linear stable junctions to reticular network like structures that are usually devoid of actin [14]. Accordingly, analysis of Rac1 and RhoA prenylation in the presence of GGTI showed different stoichiometry. Inhibition of Rac1 prenylation by GGTI was slower compared to that of RhoA (Figure 5C).

### 2.4. Nonprenylated Rho GTPases May Be Activated

**Figure 6:**
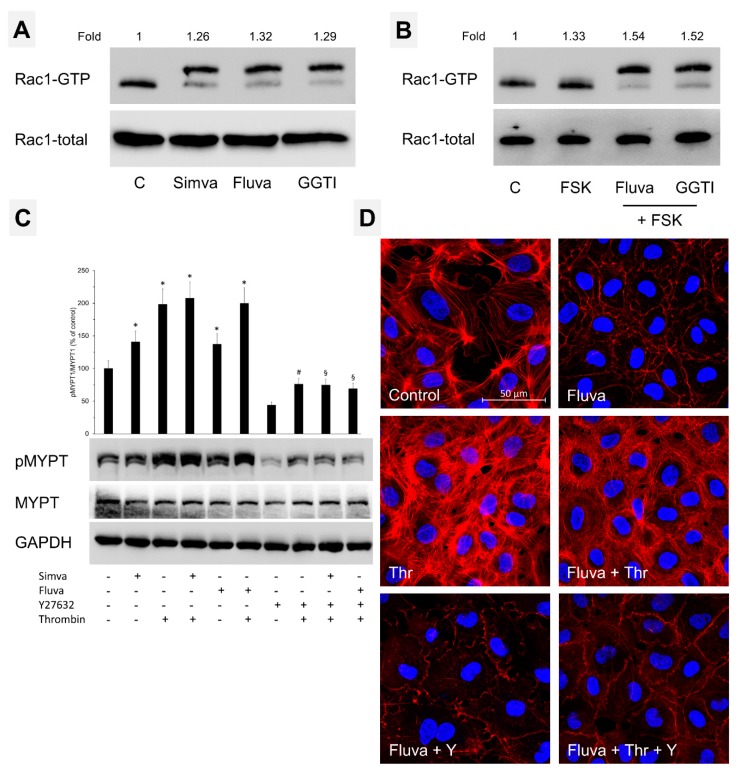
Effect of Rho GTPase prenylation on their activation. (**A**) HUVEC were treated with simvastatin (1 μM), fluvastatin (2 μM), geranylgeranyl transferase inhibitor (GGTI; 10 μM), or DMSO (C; control) for 16 h. The GTP-bound Rac1 (active) was pulled down by pulldown assay and subjected to Western blot analyses. The upper band represents the nonprenylated form of the GTPase. Representative blots from three independent experiments. The numbers above the image indicate fold change of Rac1-GTP/Rac1-total compared to control. Representative blots from three experiments. (**B**) HUVEC were treated with fluvastatin (2 μM), geranylgeranyl transferase inhibitor (GGTI; 10 μM), or DMSO (C; control) for 16 h. Afterwards the cells were treated with forskolin (FSK; 10 μM) for 10 min as indicated and the GTP-bound Rac1 (active) was pulled down by pulldown assay and subjected to Western blot analyses. The upper band represents the nonprenylated form of the GTPase. The numbers above the image indicate fold change of Rac1-GTP/Rac1-total compared to control. Representative blots from three independent experiments. (**C**) Effect of statins on thrombin-elicited RhoA/Rock activation. HUVEC were treated with simvastatin (1 μM), fluvastatin (2 μM), or DMSO (C; control) for 16 h. Afterwards, the cells were treated with thrombin, Y27632 (Rock inhibitor; 5 μM) or a combination of both as indicated. Representative Western blots of MYPT1 phosphorylation at T850 (Rock target). GAPDH and total MYPT1 was used as loading control. Representative blots from three experiments. *n* = 3; *p* < 0.05; * vs. control, ^#^ vs. Thr, ^§^ vs. statin plus Thr. (**D**) Effect of statins on thrombin-elicited actin stress fiber formation. HUVEC were treated with fluvastatin (2 μM), or DMSO (C; control) for 16 h. Afterwards, the cells were treated with Y27632 (5 μM) for 10 min followed by thrombin (0.3 I.U/mL) for 10 min as indicated. Representative immunofluorescence images (from three independent experiments) of F-actin labelled with phalloidin-TRITC. Scale bar 50 μm.

Previously, the nonprenylated forms of GTPases were considered to be inactive, therefore, we investigated whether Rac1 or RhoA in the presence of prenylation inhibitors could still be activated. As demonstrated by pulldown assay (Figure 6A), under basal conditions nonprenylated Rac1 still existed in GTP-bound form. Interestingly, treatment of HUVEC with statins or GGTI resulted in a slight increase in Rac1 activity (Figure 6A) that could be further increased with an agonist forskolin (FSK, a direct adenylyl cyclase activator) (Figure 6B). The RhoA activation was analyzed by the phosphorylation state of MYPT1, a direct substrate of RhoA-dependent kinase (Rock). As shown in Figure 6C, the basal phosphorylation of MYPT1 in the presence of simvastatin and fluvastatin was higher than the untreated control which was further enhanced when the cells were treated with thrombin and that was abolished in the presence of Rock inhibitor, Y27632 (Figure 6C). Accordingly, thrombin could induce RhoA/Rock-dependent stress fiber formation in fluvastatin-treated ECs (Figure 6D) that could completely be abolished by pretreatment with Rock inhibitor.

**Figure 7:**
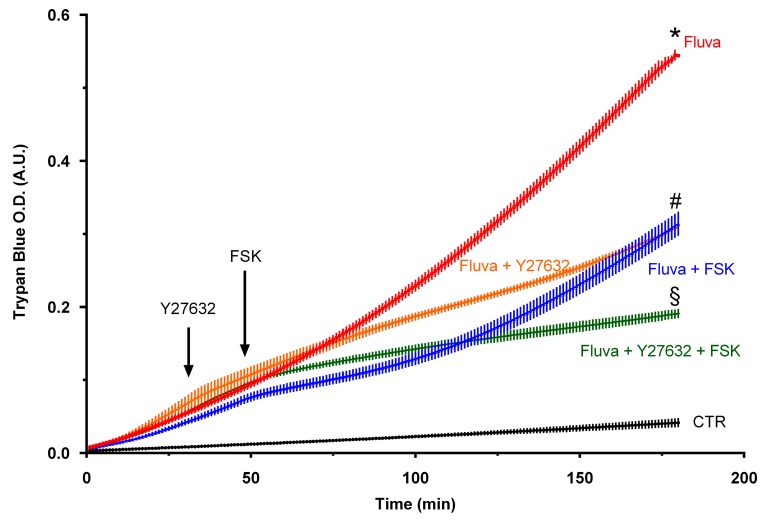
Forskolin and Rock-inhibition antagonism of statin effect on HUVEC hyperpermeability HUVECs were pretreated with fluvastatin (Fluva; 2 μM) or buffer (control) for 16 h and albumin permeability was measured. The graph represents the accumulation of albumin in the lower compartment over 3 h. Rock inhibitor Y27632 (5 μM) and forskolin (FSK; 10 μM) was added at indicated (arrows) time periods. *n* = 3; *p* < 0.05; * vs. control, ^#^ vs. Fluva alone, ^§^ vs. Fluva plus FSK or Fluva plus Y27632.

Since we observed hyperactivation of RhoA/Rock signaling in statin-treated ECs, it was investigated whether pharmacological inhibition of Rock and/or an activation of Rac1 using cAMP signaling as a maneuver could antagonize statin-induced hyperpermeability. As demonstrated in Figure 7, treatment with either Rock inhibitor (Y27632) or forskolin could reduce the permeability of HUVEC monolayers to albumin and combination of both agents had an additive effect in the late but not early phase of permeability measurement.

## 3. Discussion

EC barrier integrity is regulated by the actin-myosin based contractile machinery and actin cytoskeleton-anchored AJs that consist of VE-cadherin and catenins directly connected to the actin cytoskeleton [3]. Therefore, the stability of AJs mainly depends upon actin–cytoskeleton dynamics regulated by balanced activities of the members of Rho family of GTPases particularly RhoA and Rac1 [15]. Activation of RhoA induces the increased stress fiber formation and contraction via an activation of Rho kinase (Rock) leading to disruption of AJs. On the other hand, activation of Rac1 reorganizes actin cytoskeleton at the cell periphery thus strengthens the AJs [8]. Disruption or derangement of the activities of these GTPases under various pathophysiological conditions results in loss of EC barrier integrity [4,5]. These GTPases act as molecular switches and their activation is primarily regulated by shuttling between GDP- (inactive) or GTP-bound (active) form [9] regulated by guanine nucleotide exchange factors (GEFs) and GTPase activating proteins (GAPs) [16]. Additionally, these small GTPases are also regulated by post-translational modifications that play an important role in the control of their spatial activity. Protein prenylation is one of the well-studied post-translational modifications to be required for their membrane localization and thus spatial activity at the cellular membranes. Protein prenylation is an irreversible multistep process of addition of prenyl moieties to the terminal cysteine of CAAX motif by combined activities of an isoprenyl transferase (covalently adding prenyl group to cysteine), a protease (cleaving AAX), and a methyltransferase (ICMT; responsible for methyl esterification of the terminal cysteine) [10].

Availability of the prenyl moieties for the incorporation into GTPases is one of the rate limiting factors for protein prenylation and inhibitors of HMG-CoA reductase (generally known as cholesterol biosynthesis inhibitors or statins) are well-known and are largely employed drugs to inhibit protein prenylation to investigate the role of protein prenylation in cell biology [17,18]. Boissonneault et al., used HMG-CoA reductase inhibitor mevinolin for the first time in porcine pulmonary artery ECs while investigating the effect of oxysterols on endothelial barrier function [19] under basal conditions. The authors observed slightly increased basal albumin permeability in mevinolin-treated ECs, however, they did not investigate the effects under simulated pathological conditions. Later, the group of van Hinsbergh investigated the effect of simvastatin on HUVEC permeability. Here again, the authors observed an increased basal permeability to HRP in statin-treated cells, however, the authors show the effects of thrombin were ameliorated at very high concentration (5 μM) simvastatin [20]. In accordance, the lung research group of Joe GN Garcia has reported the protective effects of high concentration simvastatin on lung microvascular EC barrier in vitro [21] and later in a mouse lung injury model in vivo [22]. Inspired by the data from the studies of Dr. Garcia, the van Hinsbergh group has investigated whether the endothelial barrier protective effects of statins could be translated in patients. Unexpectedly, the authors have observed a prolonged ventilation therapy in patients pretreated with statins with no change in pulmonary leak index [23]. Finally, the authors conclude that prior statin-treatment does not ameliorate mildly increased vascular permeability in patients undergoing cardiovascular surgery. Accordingly, ankle and face edema are occasionally reported adverse effects of statin treatment [24,25,26,27].

In the present study, we do observe protective effects of statins on short-term treatment, however, prolonged treatment has a detrimental effect on endothelial barrier function due to effects on Rho GTPases and actin-cytoskeleton (Figure 1 and Figure 2) as discussed below. Our data (Figure 3) and available literature manifest that at least in ECs, Rho GTPases under basal conditions exist only in the prenylated form and the nonprenylated form is only observable after pharmacological intervention of the prenylation pathway. It has been reported that most of the Rho family members (e.g., RhoA, Rac1, and Cdc42) are geranylgeranylated [28,29] and it is also evident from our data that RhoA prenylation is more sensitive and responds earlier to the pharmacological inhibition of the prenylation pathway (Figure 3B and Figure 5C). This is also correlated with the first disappearance of actin stress fibers (Figure 2 and Figure 5A) that are regulated by RhoA. The peripheral actin cytoskeleton is regulated by Rac1 activity and therefore it happens to be affected at a later phase (Figure 2 and Figure 5).

Whether the nonprenylated GTPases can be activated (i.e., in GTP bound form) is controversial. However, some previous studies have shown accumulation of more GTP bound RhoA and Rac1 on treatment with various prenylation inhibitors including statins [29,30], though these studies do not investigate whether the active GTPases are prenylated. Accordingly, GGTase I knockout in mouse macrophages has resulted in an upregulation of RhoA, Rac1, and Cdc42 activities and produced high levels of proinflammatory cytokines [31,32]. Accordingly, we observed higher GTP-bound levels of Rac1 and phosphorylation of Rock substrate MYPT1 in statin-treated cells. This increased MYPT1 phosphorylation in statin-treated cells could be abrogated by a pharmacological inhibitor of Rock (Y27632) confirming an activation of RhoA/Rock. An increased basal RhoA/Rock activity under basal conditions in statins and GGTase inhibitor treated cells is translated into an increased basal permeability in these cells. Activation of cAMP signaling e.g., via FSK-mediated direct activation of adenylyl cyclase stabilizes endothelial AJs and reduces permeability via an activation of Rac1 and an inhibition of RhoA/Rock pathway [12,33,34]. In a similar line, treatment with either of the agents (FSK or Y27632) attenuated statin-induced hyperpermeability and demonstrated an additive effect when both agents are combined, suggesting that both an activation of Rac1 and inhibition of Rock is required to effectively antagonize statin-induced EC hyperpermeability. The mechanism behind basal activation of Rac1 and Rock in statin-treated or geranylgeranylation-inhibited cells still remains elusive and needs further investigation.

Actin stress fibers are regulated by the balanced activities of actin nucleation and severing proteins [35]. Rock induces actin stress fibers by phosphorylation and thus suppression of the activity of actin severing protein cofilin via activation of LIMK. Loss of prenylated RhoA/Rock signaling probably results in loss of this control on cofilin despite an increased basal Rock activity thus active cofilin can sever the actin cytoskeleton resulting in loss of stress fibers. The question arises, how thrombin can still induce stress fibers in statin-treated cells. A possible explanation for this can be the possible involvement of RhoB. RhoB has been reported to be able to switch from geranylgeranylation to farnesylation [36], is upregulated, and replaces RhoA functionality in RhoA deficient keratinocytes [37]. However, we still do not have any experimental evidence for this function and further investigation is required to elaborate this aspect.

In conclusion, inhibition of geranylgeranylation in ECs exerts a biphasic effect on endothelial barrier properties. Short-term treatment preferentially affects RhoA prenylation preserving partial Rac1 spatial activity and thus stabilizes endothelial junctions and hence barrier function. However, long-term treatment results in inhibition of both RhoA and Rac1 spatial activities resulting in collapse of actin cytoskeleton and disruption of barrier properties. Other members of the Rho family are also geranylgeranylated, and since we did not investigate these members, we cannot exclude the role of these GTPases in the actin–cytoskeleton dynamic and endothelial barrier regulation.

## 4. Materials and Methods

### 4.1. Materials

Anti VE-cadherin antibody was from Beckman Coulter (Krefeld, Germany); anti phospho MLC (S18/T19), anti phospho MYPT1 (T850), anti RhoA, anti Rac1, and anti GAPDH antibodies were from Cell Signaling Technologies (Danvers, MA, USA); Complete^®^ protease inhibitor cocktail was from Roche (Mannheim, Germany); ThinCert^®^ polycarbonate membrane filters (6-well) were from Greiner Bio-One (Frickenhausen, Germany); Benzonase^®^_,_ simvastatin, and fluvastatin were from Merck-Millipore (Darmstadt, Germany); EC basal medium plus supplement pack was from PromoCell (Heidelberg, Germany); HRP-conjugated anti-mouse IgG and -rabbit IgG antibodies were from Santa Cruz biotechnology (Heidelberg, Germany); Farnesyl pyrophosphate, Geranylgeranyl pyrophosphate, human thrombin, and phalloidin-TRITC were from Sigma (Steinheim, Germany); Pierce^®^ ECL solution, and Rac1-pulldown assay kit were from Thermo Scientific (Darmstadt, Germany); farnesyl transferase inhibitor LB42708, geranylgeranyl transferase inhibitor GGTI-298 and Y27632 were from Tocris Bioscience (Bristol, UK). All other chemicals were of the best available quality, usually analytical grade.

### 4.2. Cell Culture

The study conforms to the principles outlined in the ‘’Declaration of Helsinki’’ (*Cardiovascular Research* 1997; 35: 2–3). HUVEC were isolated from umbilical cords obtained from the gynecology department of the University hospital Giessen after approval from the ethics committee of the Medical Faculty of Justus Liebig Uiversity, Giessen (AZ 18/13 dated 28.01.2013) and informed consents from the patients. The cells were cultured as described previously [38] in complete EC culture medium (Cat # C-22010; PromoCell, Heidelberg, Germany) and used at passage 1–2. HUVEC were cultured in 6-well plates for Western blotting, 6-well filter inserts or 8-well electrode arrays for permeability assay, and 10 cm culture dishes for pulldown assay.

### 4.3. Experimental Protocols

The basal medium used in incubations was modified Tyrode’s solution (composition in mM: 150 NaCl, 2.7 KCl, 1.2 KH_2_PO_4_, 1.2 MgSO_4_, 1.0 CaCl_2_, and 30.0 *N*-2-hydroxyethylpiperazine-*N*′-2-ethanesulfonic acid; pH 7.4, 37 °C). Agents were added as indicated. Stock solutions of fluvastatin and thrombin were prepared in water, simvastatin, GGTI-298, LB42708, in DMSO and farnesyl pyrophosphate and geranylgeranyl pyrophosphate in methanol. Appropriate volumes of these solutions were added to the cells yielding final solvent concentrations ≤ 0.1% (vol/vol). Where combination of drugs was used, inhibitors were added 30–60 min before adding the statins. The same final concentrations of water, methanol, and DMSO were included in all respective control experiments.

In a set of pilot experiments the concentration–response relationships were determined to find the optimal concentration of the drugs used in the study. The drugs were used at following final concentrations; thrombin (0.3 IU/mL), simvastatin (1 μM), fluvastatin (2 μM), LB42708 (5 μM), GGTI-298 (10 μM), farnesyl pyrophosphate (2 μM), and geranylgeranyl pyrophosphate (2 μM).

### 4.4. Immunocytochemistry and Confocal Microscopy

Immunocytochemistry and confocal microscopy were performed as described previously [12]. Briefly, HUVEC were grown until confluence on glass cover slips. After treatment cells were fixed with 4% PFA, permeabilized with 0.2% Triton X-100, and blocked with blocking solution (5% BSA + 5% FCS) for 1 h. Cells were incubated with the primary antibody overnight at 4 °C and with the secondary antibody for 1 h at room temperature (RT). For actin staining the cells were stained with phalloidine-TRITC (1:50) for 1 h at RT. The cover slips were embedded in fluorescent mounting medium (CitiFluor, UK) and put onto glass slides. Images were obtained using a Zeiss LSM 710 (Zeiss; Jena, Germany) confocal microscope. The total pixel values of phalloidin and VE-cadherin were quantified using Fiji (image J) software (Version May 2017) [39].

### 4.5. Endothelial Barrier Properties

The permeability of trypan-blue-labelled albumin across HUVEC monolayers was analyzed as previously described [38].

The impedance-based endothelial barrier properties were analyzed using ECIS system (Applied Biophysics, Troy, NY, USA) according to the protocols/instructions provided by the manufacturer of the instrument.

### 4.6. Western Blotting

Western blotting was performed as described previously [38]. Blots were imaged using Fusion-FX7 imager (VWR Erlangen, Germany) and unsaturated images were analyzed using Quantity-One software (version 4.6.9, Bio-Rad, Munich, Germany). GAPDH from same gel was used as loading control for normalization of the respective protein signal.

### 4.7. GTPase Prenylation Processing Assay

Prenylation processing of GTPases was assessed with modifications from the protocol originally described by Garcia et al. [40]. The cells were harvested in ice-cold prenylation assay buffer (1% NP-40, 50 mM Tris-HCl pH 8.0, 5 mM EDTA, 1 mM phenylmethylsulfonyl fluoride and protease inhibitor cocktail). The lysate was centrifuged at 10,000× *g* to remove nuclei and the supernatants were used as cell lysates, mixed with 4× Laemmli buffer (1:4) and separated by SDS-PAGE in 12.5% polyacrylamide gels.

### 4.8. Rac1 Pulldown Assay

Rac1 pulldown assay was performed using a commercial kit (Thermo Scientific, Darmstadt, Germany) as described recently [12]. The pulled-down Rac1 protein bands (Rac1-GTP) were normalized with Rac1 bands from respective total cell lysates (Rac1-total).

### 4.9. Statistical Analysis

The data are presented as means (±S.E.M) of 3–5 experiments from independent cell preparations. The comparison between multiple groups was performed by one-way analysis of variance (ANOVA) followed by a Student–Newman–Keuls post-hoc test using Graphpad Prism 6 software (Graphpad Inc.; San Diego, CA, USA). The *p* values of ≤ 0.05 were considered statistically significant.

## Abbreviations

AJ	Adherens junctions
ECs	Endothelial cells
FPP	Farnesyl pyrophosphate
FT	Farnesyl transferase
GG-PP	Geranylgeranyl pyro phosphate
GGT	Geranylgeranyl transferase
HUVEC	Human umbilical vein endothelial cells
HMG CoA reductase	3-hydroxy-3-methyl-glutaryl-coenzyme A reductase
